# Effect of a 4 mm vs. a 6 mm Diameter Mold on the Depth of Cure of 6 Bulk-Fill Resin-Based Composites

**DOI:** 10.3390/ma18112548

**Published:** 2025-05-28

**Authors:** Anubhav Gulati, Alexandre P. Gareau, Richard B. Price

**Affiliations:** Department of Dental Clinical Sciences, Faculty of Dentistry, Dalhousie University, Halifax, NS B3H 4R2, Canada; an623775@dal.ca (A.G.); alex.gareau@dal.ca (A.P.G.)

**Keywords:** resin-based composite, depth of cure, effect of mold diameter, ISO 4049 standard

## Abstract

Dental researchers and manufacturers use the ISO 4049 standard to determine the depth of cure (DoC) of resin-based composites (RBCs). This standard uses a 4 mm diameter stainless-steel mold and subsequently divides the length of the remaining hard RBC by 2. However, the DoC values obtained using this mold have been challenged. Six bulk-fill RBCs (Tetric plus Fill, Tetric plus Flow, Tetric PowerFill, Tetric PowerFlow, Filtek One, and Aura Bulk Fill) were used to investigate the limitations of the 4 mm diameter mold used in the ISO 4049 standard when compared to a 6 mm diameter metal mold that represented the dimensions of a large cavity in a tooth. Two distinct light curing units were used. One light (Elipar S10) emitted a single peak wavelength of light, while the other (Bluephase G4) was a broad-spectrum, multiple-peak curing light. After 10 s of photocuring, the uncured RBC was immediately removed using acetone. The maximum length of the hard RBC remaining was measured and divided by two so that the effect of these two mold diameters on the DoC results could be compared. The DoC of all six RBCs tested was consistently greater in the 6 mm diameter mold (*p* < 0.0001). Sectioning revealed that the solvent-dissolved specimens had a clear internal boundary between the apparently well-cured RBC and a peripheral, solvent-resistant, “frosty” region. Using a 4 mm diameter stainless-steel mold resulted in a reduced depth of cure values compared to those obtained when the 6 mm diameter mold was used. The use of a broad-spectrum, multiple-peak LED curing light proved unnecessary for photocuring the six RBCs used in this study.

## 1. Introduction

Following international agreements to reduce the use of amalgam [1] and regulatory changes in the European Union banning the use of amalgam [2], photopolymerized resin-based composites (RBCs) have become the predominant direct restorative material. Consequently, significant research has focused on determining the polymerization metrics and how they relate to the light curing unit (LCU), the RBC, and the dimensions of the restoration. The polymerization of RBCs is frequently determined by measuring the depth of cure (DoC) according to the international ISO 4049 standard [3,4], by mechanical property testing, or by measuring the degree of conversion (DC) [4,5], where the DC refers to the percentage of monomer molecules that have been successfully converted into polymer chains during curing [5]. Many in vitro studies have shown that undercuring will produce a weaker RBC that is more prone to fracture, has a lower bond strength to the tooth, releases more chemical leachates into the mouth, and has more microleakage [6,7,8,9,10]. One in vivo study [11] placed RBC restorations in denture teeth and reported increased wear when these RBCs received inadequate light exposure.

### 1.1. Tests Measuring Depth of Cure (DoC)

In the ISO 4049 standard [3] Depth of Cure test, the RBC specimen is made in a 4 mm diameter stainless-steel mold over white filter paper. The specimen is removed immediately after light exposure, and the uncured RBC is manually scraped away using a plastic spatula [3]. The maximum length of the remaining hard, cured RBC cylinder is then measured. This maximum length is divided by two and reported as the “depth of cure” for that RBC and exposure time. This ISO standard only requires that two test specimens be made.

### 1.2. Limitations of the ISO 4049 Test

While the ISO 4049 DoC test [3] offers simplicity and ease of implementation, it makes no claims regarding correlation with the degree of conversion or the clinical performance of the RBC. In practice, cavities vary considerably in configuration and depth; few match the 4 mm diameter used in the ISO 4049 test, and none are surrounded by metal. The operator technique [12]; the type of mold material, backing, and mold diameter [13,14,15,16,17,18]; and the beam profile [19,20] are known to all affect the DoC values obtained using the ISO 4049 standard. The use of a 6 mm diameter metal mold, compared to a 4 mm diameter mold, has been reported to increase the DoC by between 6% and 8% [16]. Others have used the 6 mm diameter mold [21,22] to determine the DoC. In 2019, Erickson et al. [18] showed that RBCs that were photo-cured in a 4 mm diameter mold made from a tooth had DoCs that were significantly greater (28–35%) than those obtained from the 4 mm diameter stainless-steel mold. They reported that the ISO 4049 standard test using a 4 mm diameter metal mold was a conservative method for determining the DoC in a tooth. In addition, the DoC test described in the ISO 4049 standard only measures the maximum length of RBC remaining. Since the RBC remaining has been reported [15,18,21,23] to have a dome or “bullet-shape” appearance, this maximum length does not reflect the DoC in a tooth cavity. Also, manually removing the uncured RBC can be very subjective depending on the operator’s technique, the stiffness or sharpness of the plastic spatula, or the consistency of the RBC [12]. Instead, it would be ideal to remove uncured dental resin reproducibly using a “no-touch” approach where a solvent such as ethanol, butanone, or acetone [4,12,21,22,23,24,25,26,27,28] is used to remove the uncured resin. Using such a “no-touch” solvent dissolution technique may prevent the DoC value from being over or under-estimated.

### 1.3. Clinical Relevance of a 4 mm Diameter Metal Mold to Test DoC of Bulk-Fill RBCs

Some modern direct restoratives are designed for bulk photocuring in increments that are 4 to 5 mm thick. When considering typical cavity dimensions—with mandibular premolars averaging 7.0 mm in mesio-distal width, 7.5 mm in bucco-lingual dimension coronally, and 5.0 mm by 6.5 mm at the cervical margin [29]—testing the polymerization of a small 4 mm diameter specimen provides minimal insight into the ability of the LCU to simultaneously polymerize the mesial and distal boxes of a bulk-filled MOD restoration in a premolar tooth, let alone a larger molar tooth. The test results may be further complicated by a non-uniform distribution of light coming from the light tip of the LCU [19,20,21,23,28]. Areas of high irradiance will produce inconsistent polymerization patterns and artificially enhanced readings directly beneath the irradiance peaks, the “hot spots” of high irradiance [19,20,21,28]. It is well reported that many LCUs have these “hot spots” of high irradiance, often at the center of the light tip [19,20,21,28,30,31] and even have an uneven emission spectrum across the light tip [21,31]. Consequently, if a 4 mm diameter mold is positioned under an area of high local irradiance, or a different range of wavelengths, then the results produced may not represent the DoC that would be achieved if the RBC is positioned under a different region of the light tip [19,20,21,28].

### 1.4. Research Objectives and Hypotheses

Despite substantial evidence that mold dimensions and materials affect the DoC [13,14,15,16,17,18,20,21], the ISO 4049 standard [3] continues to use 4 mm diameter stainless-steel molds to test the DoC of all photocured RBCs. Therefore, this study examined the effect of using this 4 mm diameter mold compared to a 6 mm diameter mold [15,16,21,22,32] on the DoC measurements for six contemporary bulk-fill RBCs. Additionally, since previous research suggests multi-wavelength sources may not always be necessary [33,34,35], the performance of a single-peak-wavelength LCU was compared to a multiple-wavelength LCU.

The hypotheses tested were as follows:

(1) Increasing the mold diameter from 4 to 6 mm will not significantly affect the DoC measurements of the six RBCs tested.
(2) No significant difference will exist between DoC values obtained using single-wavelength or multi-wavelength light sources.

## 2. Materials and Methods

The DoC was measured for six different bulk-fill RBCs: Tetric plus Fill, Tetric plus Flow, Tetric PowerFill, Tetric PowerFlow, Filtek One, and Aura Bulk Fill (Table 1). The samples of each RBC light were photo-cured using a Bluephase G4 (SN1400002115, Ivoclar Vivadent, Schaan, Liechtenstein) or the Elipar S10 (1016016495, 3 M, St. Paul, MN, USA) light-emitting diode (LED) light. The Bluephase G4 light is a multiple-peak LCU manufactured by the makers of Tetric plus Fill, Tetric plus Flow, Tetric PowerFill, and Tetric PowerFlow. In contrast, the Elipar S10 light is a single-wavelength LED light manufactured by the same company that produces Filtek One.

### 2.1. LCU Radiant Power and Beam Profile

The radiant powers from the Bluephase G4 and the Elipar S10 delivered to the 4 and the 6 mm diameter test specimens were recorded five times using a 6 inch integrating sphere (Labsphere, North Sutton, NH, USA) connected to a fiber optic spectrometer (USB 4000, Ocean Optics, Dunedin, FL, USA). The measurement system was calibrated with a traceable reference standard light before testing commenced. The light output from each LCU was measured through a 4 mm or a 6 mm diameter aperture into the sphere that matched the diameter of the 4 mm and the 6 mm diameter molds used for the DoC tests. The LCU tips were centered over this aperture at 0 mm from the entrance into the integrating sphere. The irradiance distribution across the light guide tip end was then characterized using a previously described laser beam profiler technique [20] to produce color-scaled images of the irradiance distribution and radiant exposure across the end of the light guide. The laser beam analyzer camera (Model SP620U, Ophir-Spiricon, Logan, UT, USA) was focused onto the surface of a 60° holographic diffuser target (54-505; Edmund Optics, Barrington, NJ, USA). Two blue bandpass filters (34-434; Edmund Optics) and one spectrally flat reflective neutral density filter (Edmund Optics) were used to flatten the spectral response of the camera, ensuring that the violet and blue wavelengths of light were recorded equally. The beam profile images were recorded using software (BeamGage Professional version 6.21.1, Ophir-Spiricon). The BeamGage software added a 4 mm or a 6 mm diameter circle defining the beam image, and the mean radiant power values (mW) previously obtained using the integrating sphere were inserted into the BeamGage software to produce color-coded irradiance (mW/cm^2^) value images within the 4 mm or 6 mm diameter circles. These values were then multiplied by the exposure time (10 s) to produce calibrated radiant exposure images.

### 2.2. Depth of Cure (DoC) of RBCs

The uncured resin-based composites (RBCs) were placed into stainless-steel molds with either 4 mm or 6 mm diameter internal openings positioned over a white filter paper background, following the ISO 4049 standard [3]. These metal molds were 10 mm deep, and the 6 mm diameter openings represented a more clinically relevant, larger-diameter restoration than the 4 mm diameter molds. A polyester strip was positioned over each surface of the RBC before it was light-cured for 10 s, with the tip end of the LCU placed directly against the polyester surface and centered above the mold opening. The LCUs were firmly secured in position throughout the experiment, and all specimens were prepared in a random order at room temperature (approximately 22 °C), maintaining the same LCU/tip/mold orientation.

A total of 120 specimens were created (2 diameters × 6 RBCs × 5 replications × 2 LCUs). Immediately following light exposure, the polyester strips were removed, the molds were opened, and the RBC was removed. The soft, uncured RBC was left intact and not scraped away. Instead, the RBC specimen was promptly submerged in an organic solvent (Acetone, Sigma Aldrich, St. Louis, MO, USA) for 15 min at 22 °C. After removing the RBC specimens from the solvent, they were air-dried. A trained operator measured the maximum length of the specimens using digital calipers (Mitutoyo, Canada Inc., Mississauga, ON, Canada). Following the ISO 4049 standard [3], these values were divided by 2 to report the DoC in mm for each RBC. Representative specimens were cut in half longitudinally, and standardized digital photographs were taken to determine the shape of the specimens and to see if a previously documented [21,25] “frosty” layer was present at the bottom of the RBCs.

### 2.3. Statistics

The DoC results for each RBC were normally distributed. Analyses of Variance (ANOVAs) were then used to determine the impact of the RBC, LCU, and mold diameter on the length of the remaining hard RBC (StatView for Windows v.5.0; SAS Institute, Cary, NC, USA). The effects of mold diameter and the use of Bluephase G4 vs. Elipar S10 for 10 s were compared using Tukey–Kramer multiple comparison tests. All tests used a significance level of α = 0.05. The RBCs were not compared because the same exposure time (10 s) was used for all the RBCs, and this time was not always the manufacturer’s recommended exposure time.

## 3. Results

### 3.1. Radiant Power and Beam Profile

**Figure 1:**
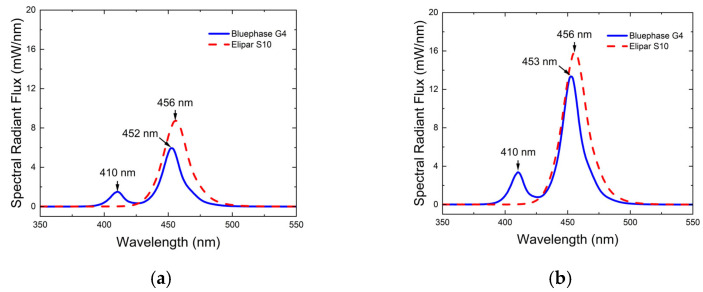
Light received by the 4 mm (**a**) and 6 mm (**b**) diameter specimens. Note the different emission spectra and the different spectral radiant flux delivered to the 4 mm diameter and the 6 mm diameter molds.

**Figure 2:**
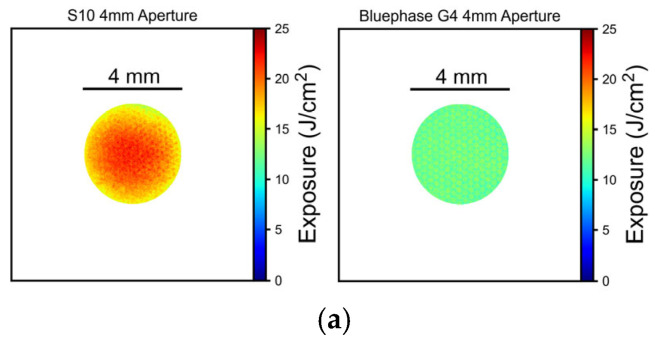

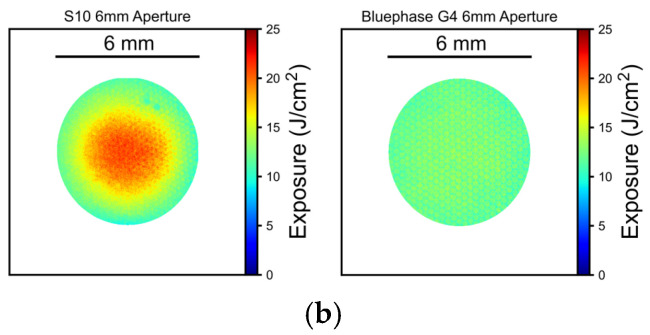
Beam profiles of the radiant exposure (J/cm^2^) received by the 4 mm (**a**) and 6 mm (**b**) diameter specimens from the S10 and the G4 lights in 10 s. Note the inhomogeneous distribution of light and energy from the S10 curing light with a “hot spot” at the center of the light tip.

Figure 1 and Figure 2 illustrate the differences in the spectral radiant powers and irradiance distributions delivered to the top surface of the 4 and 6 mm diameter molds from the two LCUs. As expected, Elipar S10 was a single-peak LCU delivering only blue light. The Elipar S10 had a region of high irradiance (mW/cm^2^) at the center that delivered a greater radiant exposure (J/cm^2^) to the center of the RBC in the mold (Figure 2). The Bluephase G4 was a multiple-peak LCU that delivered both violet and blue light, and it had a more uniform irradiance and radiant exposure beam profile.

When measured through the 4 mm diameter aperture into the integrating sphere, the radiant power from the Bluephase G4 LCU was 152 mW, while the Elipar S10’s was 55% greater at 236 mW. When measured through the 6 mm diameter aperture, the radiant power from the Bluephase G4 LCU was 345 mW, but the Elipar S10’s was only 24% greater at 427 mW. Thus, in 10 s, Bluephase G4 delivered 3.45 J, while Elipar S10 delivered 4.27 J to the 6 mm diameter specimens. However, the 4 mm diameter specimens only received 1.52 J from Bluephase G4 and 2.36 J from Elipar S10 in 10 s.

### 3.2. Remaining Cured Composite Remnant Thickness and Depth of Cure (DoC)

Depending on the test condition, after 10 s of light exposure, the mean length of hard RBC remaining ranged from 5.2 mm to 10.0 mm, or from 2.6 mm to 5 mm when divided by 2 (Table 2). See also Supplemental Figure S1 illustrating the differences in the DoC.

The ANOVA results showed that all the factors (RBC, mold diameter, and LCU) had a significant effect on the DoC. The mold diameter (*p* < 0.0001, F = 1407.90, the highest F-value) had the greatest effect. The choice of RBC also had a substantial impact (*p* < 0.0001, F = 1070.28), but this was less than the effect of the mold diameter. For all six RBCs, the 6 mm diameter mold always produced a greater depth of cure compared to the 4 mm diameter mold (*p* < 0.0001). The Tetric PowerFlow, photo-cured using the single-peak S10 light source in the 6 mm diameter mold, produced the greatest DoC of 5.0 mm.

There was a significant interaction effect between the RBC and mold diameter (*p* < 0.0001, F = 32.40). Although this interaction between the RBC and diameter was significant, Table 3 shows that this effect was weaker and had a much lower F value (F = 32.40) than the main effects of mold diameter (F = 1407.90) or the RBC (F = 1070.28). Thus, the effect of the 4 mm and 6 mm diameter mold on the DoC was not always as strong across the six RBCs. The Tukey–Kramer multiple comparison tests confirmed that there were significant differences between the RBCs and sample conditions. Although Elipar S10 produced a 10.5% to 19.0% increase in the DoC in the 6 mm diameter molds and Bluephase G4 produced a 10.8% to 22.5% increase in the DoC in the 6 mm diameter molds, overall, the Elipar S10 light produced a significantly greater DoC (*p* = 0.018) than Bluephase G4 (Table 2).

### 3.3. Bottom Surface Topography

**Figure 3:**
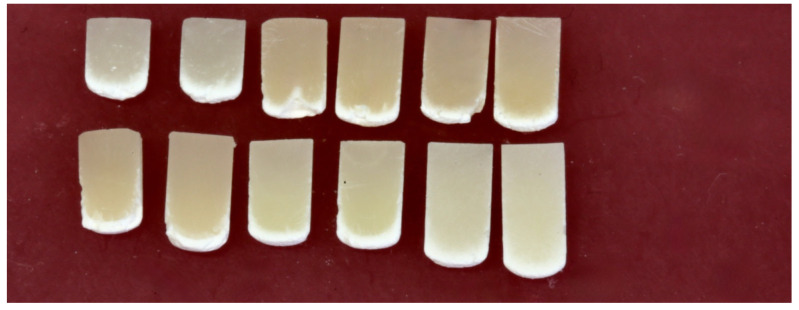
Sectioned 4 mm diameter specimens photocured using the G4 and S10 LCUs showing a cross-section of the remaining hard RBC. Note the “bullet”-shaped appearance and the “frosty” layer in all the images. The RBCs and LCUs used are in the following order from left to right: **TOP ROW:** Aura Bulk Fill G4, Aura Bulk Fill S10; Tetric plus Fill G4, Tetric plus Fill S10; Tetric plus Flow G4, Tetric plus Flow S10. **BOTTOM ROW:** Filtek One G4, Filtek One S10; Tetric PowerFill G4, Tetric PowerFill S10; Tetric PowerFlow G4, Tetric PowerFlow S10.

**Figure 4:**
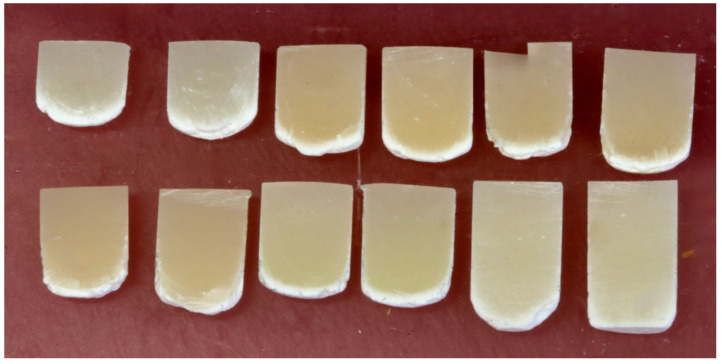
Sectioned 6 mm diameter specimens photocured using the G4 and S10 LCUs showing a cross-section of the remaining hard RBC. Note the “bullet”-shaped appearance and the “frosty” layer in all the images. The RBCs and LCUs used are in the following order from left to right. **TOP ROW:** Aura Bulk Fill G4, Aura Bulk Fill S10; Tetric plus Fill G4, Tetric plus fill S10; Tetric plus Flow G4, Tetric plus Flow S10. **BOTTOM ROW:** Filtek One G4, Filtek One S10; Tetric PowerFill G4, Tetric PowerFill S10; Tetric PowerFlow G4, Tetric PowerFlow S10.

When sectioned longitudinally in half, the remaining hard RBC had an unsymmetrical bottom surface profile (see Figure 3 for the 4 mm diameter specimens and Figure 4 for the 6 mm diameter specimens). The ends of the RBC specimens had a distinct “bullet” shape, and a “frosty” layer of partially solvent-resistant RBC was present at the bottom of all the RBCs.

## 4. Discussion

This investigation required only one RBC to have a significant difference in the DoC achieved in the 4 mm and 6 mm diameter molds to question the results from the current ISO 4049 standard. Instead, all six RBCs achieved a 10.5 to 22.5% increase in the DoC in the 6 mm diameter molds (Table 2). This was very significantly greater than the DoC results achieved in the 4 mm diameter molds (*p* < 0.0001). Thus, the first research hypothesis was rejected. Furthermore, the Elipar S10 single-peak-wavelength LCU that delivered greater total energy with a “hot spot” of high irradiance at the center (Figure 4) consistently produced greater DoC values (*p* = 0.018) compared to the multiple-peak wavelength LCU that had a more uniform output distribution (Table 2); thus, the second hypothesis that there would be no difference between the LCUs was rejected.

As illustrated in Figure 3 and Figure 4, except for the Tetric PowerFlow that had been exposed to the S10 light for 10 s in the 6 mm diameter mold, the remaining RBCs were all dome-shaped, and a “frosty” layer was present at the bottom (Figure 4). Had the 6 mm diameter metal mold been longer, the DoC of the Tetric PowerFlow would likely have been slightly longer in the 6 mm diameter mold. A similar dome or “bullet-shape” appearance and increased hardness at the center of RBC specimens compared to the outer regions have been reported previously [14,15,17,18,19,21,23]. Previous studies have also reported that the hardness of the RBC photocured in opaque molds was substantially lower at or near the walls of the mold than at the center and that the depth of cure was also dependent on the diameter of the mold [14,15,18,19,21]. While this may be due to the opaque nature of the mold [14], this effect may also be attributed to the fact that not all surface areas of the specimen received the same radiant exposure from the LCU [19]. This was illustrated in Figure 2, where the S10 light delivered more energy to the RBC at the center of the mold. This inhomogeneous distribution of light across the light tip may be the reason why the “bullet-shaped” curing pattern was observed in the RBCs [23], but since the G4 delivered a homogeneous distribution of light, this “bullet-shaped” curing pattern may also be due to the thermal mass of the stainless-steel mold that reduced the temperature increase in the RBC that was close to the metal walls as it polymerized [23]. This reduced the beneficial effect of the exothermic temperature increases that occurred during polymerization.

The ISO 4049 standard only requires a sample size of 2. The present study used a sample size of 5, and the statistical power values were 1.0, confirming that the sample size and statistical tests were highly effective in detecting differences. As shown in Figure 3 and Figure 4 and Table 2 and Table 3, the mold diameter caused a 10.5 to 22.5% increase in the DoC in the 6 mm diameter molds and was the most significant factor that affected the length of the remaining hard RBC (*p* < 0.0001). Despite the large volume increase in the total amount of material used in the 6 mm diameter mold compared to the 4 mm diameter mold (from 125.7 mm^3^ to 282.7 mm^3^), when the RBCs were exposed using the same 10 s exposure time, the length of the remaining hard RBC remaining always increased as the mold diameter increased from 4 mm to 6 mm. This may be because Bluephase G4 delivered 3.45 J, whereas Elipar S10 delivered 4.27 J to the 6 mm diameter specimens in 10 s. In contrast, the 4 mm diameter specimens only received 1.52 J from Bluephase G4 and 2.36 J from Elipar S10 in 10 s. Thus, although the volume of the 6 mm diameter mold was greater, the greater DoC achieved in the 6 mm diameter mold is likely because the RBC in the 6 mm diameter mold received more energy from both LCUs. The fact that more energy was delivered from the single-peak Elipar S10 light also explains why the Elipar S10 produced a significantly greater DoC (*p* = 0.018) compared to the multi-peak G4. This observation that the Elipar S10 LCU delivered 55% more energy than the Bluephase G4 through the 4 mm aperture, but only 24% more energy through the 6 mm aperture, highlights the irradiance beam’s non-uniformity delivered by Elipar S10. This LCU has now been replaced by the Elipar Deep Cure, which has a more uniform beam profile.

This study did not conduct a chemical analysis of the photoinitiators used in the six RBCs tested. However, four of these RBCs, Tetric plus Fill, Tetric plus Flow, Tetric PowerFill G4, and Tetric PowerFlow, are made by Ivoclar. These RBCs use a combination of camphorquinone and Ivocerin [36], while the manufacturers of Aura Bulk Fill and Filtek One state that these RBCs use only camphorquinone [37,38]. Filtek One, Tetric PowerFlow, Tetric PowerFill, and Tetric plus Flow produced test specimens that were at least 4 mm long in the 6 mm diameter mold after a 10 s exposure using the single-peak Elipar S10 light (Table 2). This result confirms other reports that a multiple-wavelength peak LCU is not always required [33,34,35].

In clinical practice, cavities vary in shape and depth, and multiple variables influence the DoC, including the presence of adjacent walls and the cavity size. While the ISO 4049 standard, which uses a 4 mm diameter mold is a simple screening method, it only measures the maximum length of hard RBC remaining. The resulting depth of cure values are, therefore, highly dependent upon the operator [12], the maximum length measurement, and the beam homogeneity of the LCU. Given the “bullet-shaped” appearance of the RBCs, it is important to report the shape of the hard RBC remaining. Placing the RBC in acetone for 15 min was considered less aggressive than the Acetone Shake Test [12,27], where the RBC is placed in acetone in an amalgamator and vibrated for 15 s. This vibration jostles the RBC and mechanically removes some of the RBC. Instead, this study’s 15 min immersion “no-touch” technique removed the uncured RBC without any physical contact. This eliminated the operator’s subjective judgment and any inconsistent performance when scraping away the uncured RBC [12,21,22,25,26].

When the solvent-dissolved RBC specimens were sectioned, a clear internal boundary between the RBC that was apparently well-cured, and the peripheral, porous, solvent-resistant polymer could be seen. This region has been previously characterized as “frosty” [21,25]. This “frosty” region followed the contour of the hard bottom surface of the RBC (Figure 3 and Figure 4) and was approximately 1.0 to 1.5 mm wide. Its width appears to be visually similar to the 2.2 mm difference between the length of hard RBC that remains after the soft RBC has been scraped away and the region where clinically relevant differences in the RBC properties have been reported to begin [24]. As illustrated in Figure 3 (the 4 mm diameter specimens) and Figure 4 (the 6 mm diameter specimens), this “frosty” region never penetrated into the first half of the RBC closest to the light. The presence and configuration of this porous region suggest that the method used in the ISO 4049 standard [3] of reporting half the maximum length of the remaining hard RBC as the depth of cure (DoC) may provide inaccurately short DoC values.

Since the manufacturer’s recommended exposure times were not always used, the study did not compare the DoC of the RBCs. When using this 10 s exposure, Aura Bulk Fill produced the shortest DoC of 2.6 to 3.1 mm, depending on the mold and LCU used (Table 3). This was likely because Aura Bulk Fill and Filtek One received only half of their recommended exposure times (Table 1). This result supports the conclusions of a previous study [22] that reported the importance of following the instructions for use provided by the manufacturer of the RBC, not those of the manufacturer of the LCU.

While the results obtained using a 4 mm diameter metal mold can provide a standard laboratory test, its relevance in the clinical setting where most cavities are larger than 4 mm in diameter is uncertain [39]. Since the RBCs photocured in the 6 mm diameter mold consistently yielded higher DoC values, this finding supports Erickson et al.’s [18] conclusion that modifications to the current ISO 4049 standard [3] are necessary. While the influences of the mold’s thermal mass, volume, and solvent immersion duration have yet to be fully determined, using a 6 mm diameter mold may provide outcomes that are more clinically relevant than the 4 mm diameter mold currently specified in the ISO 4049 standard.

The purpose of this study was to demonstrate and confirm that there were significant differences in the DoC values between the 4 and 6 mm diameter molds. The 6 mm diameter mold has been used previously by others [15,16,21,22] and was chosen to represent a large restoration in a premolar tooth. A wider, e.g., 7 mm diameter mold, would have been wider than the active diameter of some light guides [19,28], and, thus, the RBC in the mold would not have received direct light. Thus, a 6 mm diameter mold appears to be the largest that can be used.

Although this study found that the 6 mm diameter mold always produced a greater depth of cure in the six bulk-fill RBCs tested, not all available RBCs were tested. Additionally, a standard 10 s exposure time was used for all RBCs to minimize the number of variables in the study. This time was shorter than the manufacturer’s recommended exposure time for Filtek One and Aura Bulk Fill, which was why the DoC between the RBCs was not compared. Future studies into the DoC of RBCs could use a mold that better simulates a human tooth’s shape, thermal properties, and light transmission characteristics. Future studies should develop specific guidelines for identifying the ideal solvent and the optimal duration of solvent exposure. Given the “bullet-shaped” appearance of the RBCs, future studies should also report the shape of the hard RBC remaining. Ongoing microhardness tests and degree of conversion analyses are attempting to identify the nature of the polymerization along the “frosty layer” at the bottom of the solvent-dissolved RBCs.

## 5. Conclusions

This study showed that

1. The 6 mm diameter metal molds consistently produced greater depth-of-cure results than standard 4 mm diameter configurations specified in the ISO 4049 standard (*p* < 0.0001).
2. The single-peak-wavelength LCU provided equivalent or superior performance compared to the multiple-peak-wavelength LCU tested in this study.
3. The RBC polymerized in the metal molds consistently exhibited a dome-shaped morphology with a greater DoC at the center compared to the peripheral regions that were close to the metal walls.

## Figures and Tables

**Table 1:** Manufacturers’ location, lot numbers, shades, initiators, and the minimum irradiance values recommended by the manufacturers [36,37,38] of the bulk-fill RBCs used in the study.

RBC	Manufacturer	Lot Number	Shade	Initiator	Minimum Irradiance and Recommended Wavelength Range
Tetric plus Fill	Ivoclar Vivadent, Schaan, Liechtenstein	YM0206	A3	Camphorquinone plus Ivocerin	900–1400 mW/cm^2^ for 10 s in the 400–500 nm range
Tetric plus Flow	Ivoclar Vivadent, Schaan, Liechtenstein	YM0210	A3	Camphorquinone plus Ivocerin	900–1400 mW/cm^2^ for 10 s in the 400–500 nm range
Tetric PowerFill	Ivoclar Vivadent, Schaan, Liechtenstein	Z06955	IVA	Camphorquinone plus Ivocerin	900–1400 mW/cm^2^ for 10 s in the 400–500 nm range
Tetric PowerFlow	Ivoclar Vivadent, Schaan, Liechtenstein	Z06P1N	IVA	Camphorquinone plus Ivocerin	900–1400 mW/cm^2^ for 10 s in the 400–500 nm range
Filtek One	3 M, St. Paul, MN, USA	4867A3	A3	Camphorquinone	1000–2000 mW/cm^2^ for 20 s in the 400–500 nm range
Aura Bulk Fill	SDI, Bayswater, Victoria, Australia	1222869	Universal	Camphorquinone	High-power LED curing light for 20 s in the 460–480 nm range

**Table 2:** Mean length (mm) divided by 2 of the hard resin-based composite (RBC) remaining, depending on the mold diameter and LCU used. Shaded cells represent DoC values that were greater than 4 mm. The increase (%) when the 6 mm diameter mold was used compared to the 4 mm diameter mold is also reported.

RBC, Mold Diameter and LCU	Length (mm)	S.D.	Increase (%) Using the 6 mm Diameter Mold
PowerFlow, 6 mm, S10	5	0.01	19.0%
PowerFlow, 6 mm, G4	4.9	0.02	22.5%
Filtek One, 6 mm, S10	4.2	0.09	10.5%
PowerFlow, 4 mm, S10	4.2	0.08	
PowerFill, 6 mm, S10	4.1	0.09	17.1%
Tetric plus Flow, 6 mm, S10	4.1	0.04	13.9%
PowerFlow, 4 mm, G4	4	0.04	
Filtek One, 4 mm, S10	3.8	0.03	
PowerFill, 6 mm, G4	3.8	0.08	15.2%
Tetric plus Fill, 6 mm, S10	3.8	0.08	11.8%
Tetric plus Flow, 6 mm, G4	3.8	0.05	11.8%
Filtek One, 6 mm, G4	3.7	0.04	10.8%
Tetric plus Fill, 6 mm, G4	3.6	0.13	12.5%
Tetric plus Flow, 4 mm, S10	3.6	0.12	
PowerFill, 4 mm, S10	3.5	0.12	
Tetric plus Fill, 4 mm, S10	3.4	0.11	
Tetric plus Flow, 4 mm, G4	3.4	0.03	
Filtek One, 4 mm, G4	3.3	0.04	
PowerFill, 4 mm, G4	3.3	0.05	
Tetric plus Fill, 4 mm, G4	3.2	0.06	
AuraBulk, 6 mm, S10	3.1	0.07	14.8%
AuraBulk, 6 mm, G4	2.9	0.07	11.5%
AuraBulk, 4 mm, S10	2.7	0.08	
AuraBulk, 4 mm, G4	2.6	0.04	

**Table 3:** ANOVA results showing the effects of the resin-based composite (RBC), the mold diameter, and the LCU on the length of hard RBC remaining.

Factor	DF	Sum of Squares	Mean Square	F-Value	*p*-Value	Power
RBC	5	29.35	5.87	1070.28	<0.0001	1.0
Mold Diameter	1	7.72	7.72	1407.90	<0.0001	1.0
LCU	1	1.88	1.88	342.79	<0.0001	1.0
RBC*Mold Diameter	5	0.89	0.18	32.40	<0.0001	1.0
RBC*LCU	5	0.32	0.06	11.67	<0.0001	1.0
Mold Diameter* Light	1	0.00	0.00	0.37	0.55	0.09
RBC*Mold Diameter* LCU	5	0.06	0.01	2.26	0.05	0.71
Residual	96	0.53	0.01			1.0

## Data Availability

The data are available from the authors upon request.

## Supplementary Materials

The following supporting information can be downloaded at: https://www.mdpi.com/article/10.3390/ma18112548/s1, Figure S1: Effect of Mold Diameter on Depth of Cure.

## Abbreviations

The following abbreviations are used in this manuscript:

LCU	Light Curing Unit
RBC	Resin-based composite
DoC	Depth of Cure
