# Supplementary figures and images for: Effect of a 4 mm vs. a 6 mm Diameter Mold on the Depth of Cure of 6 Bulk-Fill Resin-Based Composites
Source: Materials (Basel). 2025 May 28;18(11):2548. doi: 10.3390/ma18112548 (PMC12155904; doi:10.3390/ma18112548)

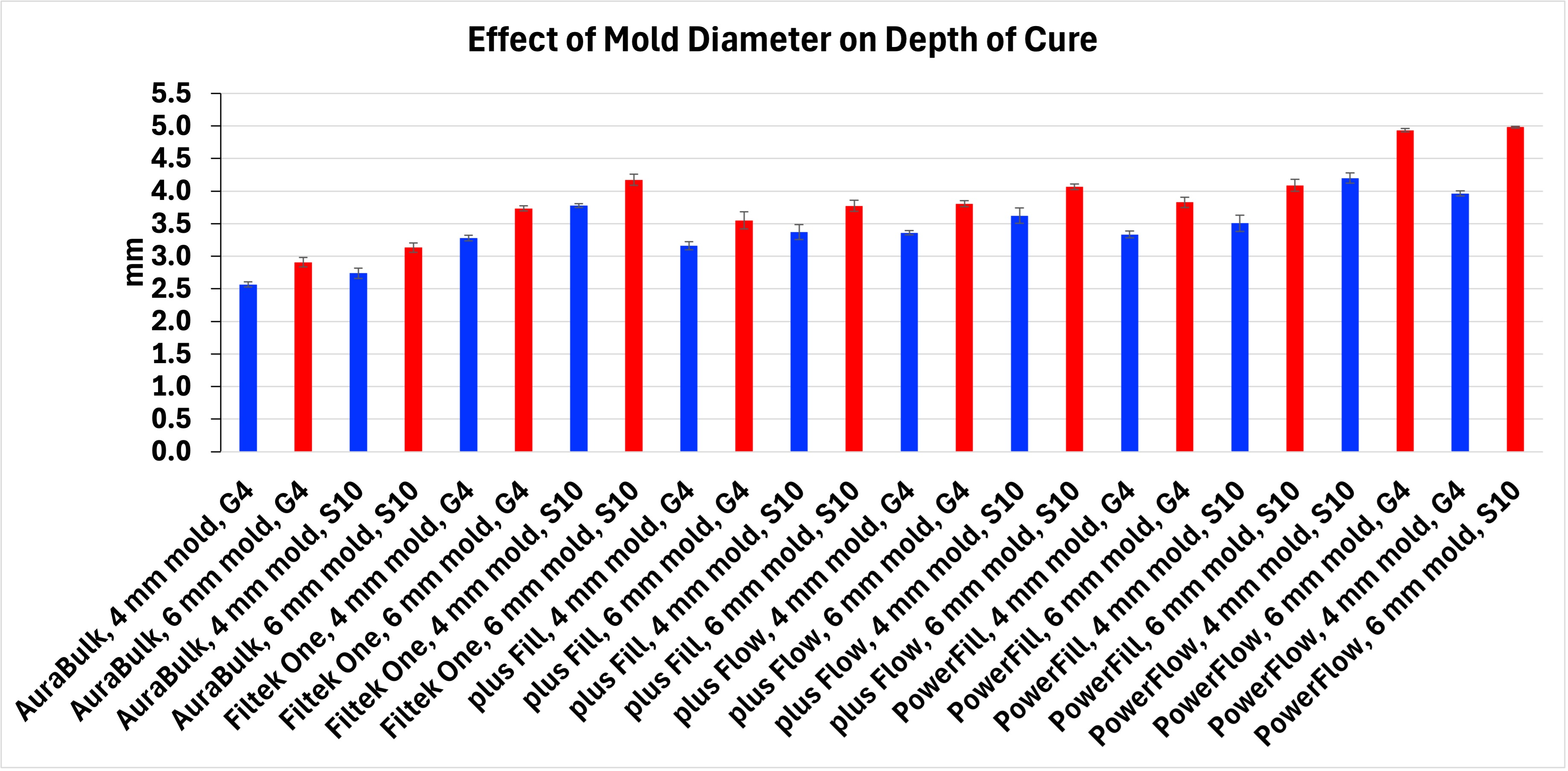

Supplement: Supplementary file 1 [file materials-18-02548-s001.zip › materials-3633904-supplementary.tiff]
